# Image-based honey bee larval viral and bacterial diagnosis using machine learning

**DOI:** 10.1038/s41598-025-16261-5

**Published:** 2025-08-21

**Authors:** Duan C. Copeland, Brendon M. Mott, Oliver L. Kortenkamp, Robert J. Erickson, Nathan O. Allen, Kirk E. Anderson

**Affiliations:** 1 USDA-ARS Carl Hayden Bee Research Center, 2000 E. Allen Rd, Tucson, AZ 85719 USA; 2 Department of Entomology and Center for Insect Science, University of Arizona, Tucson, AZ 85721 USA

**Keywords:** Deep learning, Artificial intelligence, Honey bee, Transfer learning, EFB, Virus, Larvae, Antibiotics, Antimicrobial resistance, Infectious-disease diagnostics, Molecular biology, Microbiology, Virus-host interactions

## Abstract

Honey bees are essential pollinators of ecosystems and agriculture worldwide. With an estimated 50–80% of crops pollinated by honey bees, they generate approximately $20 billion annually in market value in the U.S. alone. However, commercial beekeepers often face an uphill battle, losing anywhere from 40 to 90% of their hives yearly, often by brood diseases caused by bacterial, viral, and fungal pathogens. Accurate diagnosis of brood diseases, especially distinguishing European Foulbrood (EFB) from viral infections with a superficial resemblance to EFB (EFB-like disease), remains challenging. Incorrect diagnoses often lead to prophylactic antibiotic treatment across entire apiaries, exacerbating antibiotic resistance, disrupting native gut microbiota, and increasing susceptibility to opportunistic pathogens. Correct field diagnosis of brood disease is challenging and requires years of experience to identify and differentiate various disease states according to subtle differences in larval symptomology. To explore the feasibility of an image-based AI diagnosis tool, we collaborated with apiary inspectors and researchers to generate a dataset of 2,759 honey bee larvae images from Michigan apiaries, molecularly verified through 16 S rRNA microbiome sequencing and qPCR viral screening. Our dataset included EFB cases and viral infections (ABPV, DWVA, and DWVB), which were augmented to 8,430 and 8,124 images, respectively. We leveraged transfer learning techniques, fine-tuning deep convolutional neural networks (ResNet-50v2, ResNet-101v2, InceptionResNet-v2) pre-trained on ImageNet to discriminate between EFB and viral infections. Our proof-of-concept models achieved 73–88% accuracy on the training/validation sets. When tested on an independent dataset from Illinois containing additional viral pathogens not present in training data, the models showed higher accuracy for EFB (72–88%) than viral infections (28–68%), highlighting both the promise and current limitations of this approach. Implementing AI-based diagnostic tools can reduce unnecessary antibiotic treatments and help maintain the microbiome integrity critical to colony health. However, expanding training datasets to include all major pathogens, healthy larvae, and diverse geographic regions will be essential for developing field-ready diagnostic tools.

**Supplementary Information:**

The online version contains supplementary material available at 10.1038/s41598-025-16261-5.

## Introduction

Honey bees (*Apis mellifera* L.) are social, colonial insects that are often managed at densely packed sites called apiaries. Unlike most livestock, U.S. honey bee hives are transported across the country for pollination services^1,2^. Honey bee populations in the United States are experiencing unsustainable losses, with each of the last two years representing greater than 50% annual loss in managed commercial colonies^3,4^. Colony loss is multifactorial, often involving combinations of environmental stress and disease agents^5–8^. Many of these pathogens primarily affect honey bee larvae, leading to what are collectively known as brood diseases^6,9,10^. These pathogens impact colony survival as colony success depends on perpetual worker production. The continuous overlap of new and old worker generations facilitates learning, task specialization, and host-microbial evolution^11–13^. The integrity and continuation of the worker group are essential to construct a built structure (hive), support massive egg production by the queen, ensure continuous larval feeding, and maintain the healthy division of labor within the colony.

A large number of brood diseases are caused by various bacterial, viral, and fungal agents^6,10,14^. Field diagnosis of brood disease based on symptomology requires the trained gestalt of an experienced apiarist or master beekeeper. Of greatest overall concern, the application of antibiotics to treat unidentified brood disease is often ineffective, instead promoting the evolution of antimicrobial resistance genes^15^. Recently, our lab explored both symptomology and microbial succession of diseased larvae, photographing and sampling healthy and diseased larvae identified in the field as the bacterial pathogen European Foulbrood (EFB) or EFB-like (virus) from over 30 apiaries across the state of Illinois^14^. The significant relationship between larval symptomology and molecular results revealed two very different disease states with similar symptomology: EFB and disease caused by various viral agents. Typically, apiary inspectors and beekeepers are unable to give a specific diagnosis, requiring reference to molecular data and other colony-level factors like parasite (Varroa mite) load^8,16,17^. Our recent results suggest that the microbiome composition of larvae is often disturbed by beekeeper-applied antibiotics, increasing the susceptibility of larvae to opportunistic disease^14^. Consistent with long-term effects of the antibiotic oxytetracycline, both healthy and diseased larvae from symptomatic colonies show a significant deficit of healthy microbiota, including gram-positive bacteria relative to healthy apiaries. Under treatment conditions, gram-negative species known to carry antibiotic resistance genes were the only bacteria found blooming in healthy and diseased larvae^14^. Various tetracycline-resistant bacteria have already been identified in honey bees across the U.S^10^. and globally^18–20^. The overuse of antibiotics over the long term selects for antibiotic resistance^21,22^, potentially rendering hosts more susceptible to opportunistic and antibiotic-resistant microbes, including Deformed Wing Virus (DWV), Acute Bee Paralysis Virus (ABPV), and *Serratia marcescens* that are significantly elevated in larvae with advanced viral disease^14^.

To address these challenges, we propose leveraging machine learning (ML) and artificial intelligence (AI) techniques to revolutionize the diagnosis and management of honey bee brood diseases and colony health^23^. ML/AI projects are flexible and have already been successfully implemented to solve many image-based challenges in honey bees, such as subspecies identification via wing geometric morphometrics^24,25^monitoring honey bee pollen foraging behavior^26^detecting *Varroa destructor* parasitic mites^27^and counting honey bee capped brood cells^28^. Here, we developed a proof-of-concept AI-driven diagnostic system capable of differentiating between bacterial and viral infections in honey bee brood. Using molecularly-verified images from Michigan apiaries, we trained models to distinguish between EFB and viral infections (ABPV, DWV, and DWVB), then tested generalization on an independent dataset from Illinois. Our objectives were to: (1) develop and validate deep learning models for distinguishing between EFB and viral infections using transfer learning approaches, (2) evaluate model performance, and (3) demonstrate explainable AI techniques as tools for future model validation and development.

## Methods

### Sample collection, nucleic acid extraction, and disease verification

Field samples were collected from honey bee colonies across multiple states through collaboration with state apiary inspectors, commercial beekeepers, and research institutions. Colonies designated as diseased by apiary inspectors were photographed at both the frame and individual larval level (Figure S1 and S2 for example images). Digital photographs were taken of both symptomatic and asymptomatic larvae, with identification tags placed for sample tracking. From each apiary, we sampled both healthy and diseased colonies, targeting larvae across multiple developmental stages (3rd, 4th, and 5th instar). For molecular verification of disease status, we collected 20–30 larvae per symptomatic colony, sampling four replicates for each of three larval stages from both healthy and diseased hives within the same apiary.

We employed a dual-extraction protocol to obtain both RNA (host and virus) and DNA (bacterial and fungal) from individual larvae. Samples were processed using a modified protocol combining ThermoScientific GeneJET RNA and DNA extraction kits (Thermo Fisher Scientific, Waltham, Massachusetts, United States). DNA/RNA were extracted from larvae with known symptomology and photographed condition. The larval microbiota was analyzed using 16 S rRNA gene sequencing, while viral screening was conducted via real-time quantitative PCR for known viral and EFB. Samples were pooled by hive and phenotype for initial screening, followed by targeted studies on individual samples using amplicon sequencing of 16 S rRNA regions to characterize microbial communities.

Briefly, whole larvae were physically lysed in bead-beating tubes containing 0.1 mm beads and 600µL TE buffer supplemented with 2% β-mercaptoethanol. Following an initial 30-second bead-beating step, 300µL of lysate was immediately removed for RNA extraction and stabilized in an equal volume of RNA lysis buffer. The remaining lysate underwent an additional 90-second bead-beating step for optimal bacterial DNA extraction. For DNA extraction, the lysate was treated with lysozyme at 37 °C, followed by proteinase K digestion at 56 °C. After RNase treatment and ethanol precipitation, DNA was purified using spin columns and eluted in 100µL elution buffer. RNA was extracted from the fractionated sample, with subsequent column purification and elution in 100µL nuclease-free water. We created a cDNA template from the purified RNA fraction. Briefly, RNA was converted into cDNA with Thermo Scientific RevertAid First Strand cDNA Synthesis Kit (Thermo Fisher Scientific, Waltham, Massachusetts, United States) following manufacturer’s instructions. The cDNA was used to screen for European Foulbrood (EFB) and honey bee viral pathogens by pooling ten larvae from either healthy or diseased hives in the same apiary. The screened viruses were Acute Bee Paralysis Virus (ABPV), Black Queen Cell Virus (BQCV), Chronic Bee Paralysis Virus (CBPV), Deformed Wing Virus (DWV-A and DWV-B), Israeli Acute Paralysis Virus (IAPV), Kashmir Bee Virus (KBV), and Sacbrood Virus (SBV. A viral hive diagnosis was given based on the no EFB being detected in the pools. A list of primers is provided in Table S1.

### DNA sequencing and 16 S rRNA gene community analysis

Using the DNA, we quantified total bacterial abundance for singular whole larvae using a quantitative PCR (qPCR) assay of the 16 rRNA gene^29^. We created a standard curve using a tenfold serial dilution series of a plasmid standard containing a full-length *Escherichia coli* 16S rRNA gene. We amplified a 466 bp fragment in the V3–V4 region of the 16S rRNA gene using universal primer pair (S-D-Bact-0341- b-S-17 5’– CCTACGGGNGGCWGCAG − 3’ and S-D-Bact-0785-a-A-21 5’– GACTACHVGGGTATCTAATCC − 3’. PCR reactions were performed in triplicate on a BioRad CFX96 (Biorad, Hercules, California, US) as follow: 12 µl reactions containing 9 µl of iTaq Universal SYBR Green Supermix (BioRad, Hercules, California, US), 0.5 µl forward primer,0.5 µl reverse primer, and 2 µl of DNA template. The cycling conditions were 95 °C for 3 min followed by 40 cycles of 95 °C for 10 s and 60 °C for 60 s. The qPCR results were expressed as the total number of 16 S rRNA gene copies per DNA extraction (200 µl volume elution).

AZENTA Life Sciences (https://www.azenta.com) performed the sequencing using a MiSeq (v3 PE-300 kit) following the manufacturer’s DNA library preparation protocol. We targeted the V3-V4 region for amplification of the 16 S rRNA gene using PCR primers (341 F 5′-CCTACGGGNGGCWGCAG-3′; 805R 5′-GACTACHVGGG TATCTAATCC-3′). The sequence data for this study have been deposited in GenBank, Sequence Read Archive no. as PRJNA1285920.

16S rRNA gene sequence data were processed using MOTHUR v.1.44.34351 according to previously published protocols^30^. Briefly, paired end reads were joined using the “make.contigs” command. We used the SED command in UNIX to remove the first and last five nucleotides from each sequence. Sequences were then screened to remove ambiguous bases, using the “screen.seqs” command. Unique sequences were generated using the “unique.seqs” command. A count file containing group information was generated using the “count.seqs” command. Sequences were aligned to the BEExact database^31^ using the “align.seqs” command. Sequences were filtered to remove overhangs at both ends and gaps using “filter.seqs”. The “unique.seqs” command was ran again to remove new redundancies from filtering. A precluster step using “pre.cluster” was performed followed by the “chimera.uchime” command^32^ to remove chimeric sequences. Sequences not of bacterial origin (fungi, archaea, mitochondria, and chloroplasts) were removed using the “remove.seqs” command. All unique sequences with one or two members (single/doubletons) were removed using the AWK command in UNIX. A distance matrix was constructed for the aligned sequences using the “dist.seqs” command. Sequences were classified at the unique level with the BEExact database using “classify.seqs” command. Unique sequences were then merged at the species-level with the “merge.otus” command.

### Image dataset preparation

High-resolution photographs of diseased larvae were closely cropped to present 1–3 diseased larvae, reflecting the variable ways beekeepers might photograph suspected disease in field conditions. This approach, rather than standardizing to single centered larvae, was chosen to accommodate different photographic compositions and the clustered disease patterns often observed in brood frames. Images were collected using various cameras and by operators with different skill levels, intentionally introducing variability that reflects real-world usage scenarios. The initial training dataset consisted of molecularly verified cases of EFB infection (*n* = 1,405 images) and viral infection (*n* = 1,354 images), providing a well-balanced dataset. The viral infection category included larvae infected with co-infections of Acute Bee Paralysis Virus (ABPV) and Deformed Wing Virus (DWV) A and B, diagnosed from the absence of EFB in colony pools Images were preprocessed using standard computer vision techniques including resizing to 224 × 224 pixels or 299 × 299 pixels depending on the transfer learning model used. After normalization, we employed image augmentation through rotation, flipping, and controlled brightness and contrast adjustments to expand the training set with 8,430 EFB images and 8,124 viral infection images, maintaining balanced representation of both confirmed EFB infection and confirmed viral infection cases (Table S2). The brightness and contrast adjustments were designed to simulate varying field conditions including different lighting environments and camera settings that beekeepers might use, while preserving the diagnostic color relationships found within the hive.

### Model development

We implemented our deep learning models using TensorFlow^33^leveraging transfer learning with pre-trained model architectures including ResNet-50v2, and ResNet-101v2, and InceptionResNet-v2. All models were pre-trained on the ImageNet dataset (ILSVRC-2012) containing 1.2 million images across 1,000 categories. The base models were fine-tuned on our specialized dataset of honey bee brood disease images (Table S3). The base models were fine-tuned on our specialized dataset of honey bee brood disease images. Training was conducted using a step-wise decreasing learning rate strategy to optimize model convergence and reduce overfitting, with the dataset split into 70% training, 15% validation, and 15% test sets.

### Model validation and performance analysis

Model performance was assessed using standard metrics including accuracy, precision, recall, and F1 score (Fig. 2). To evaluate real-world generalization, we tested our models on an independent dataset collected from Illinois apiaries in 2019, consisting of 3,184 molecularly-verified EFB images and 2,981 viral infection images. Here, the viral disease pools indicate the colonies were a mix of co-infections of DWVA, DWVB, BQCV, ABPV, and IAPV. This geographically and temporally distinct test set was not used during training or validation, providing a rigorous assessment of model performance. Confusion matrices were generated to analyze the distribution of correct classifications and misclassifications between EFB and viral infections.

For misclassified cases and to evaluate model interpretability, we performed detailed analysis using explainable AI techniques to understand the factors contributing to incorrect predictions. This included examination of saliency maps and comparison with molecular diagnostic results to identify patterns in classification errors (Fig. 3).

### Statistical analysis

In the microbiota analysis, the 24 most abundant species designations were normalized by absolute abundance by first calculating the relative amount of each species based on raw read count per sample. Absolute species abundance was calculated as the product of species relative abundance and 16 S rRNA gene copies determined with BactQuant. 16 S rRNA gene copy number was assigned to each species based on their closest taxonomic representative^34^. A sum of outstanding species were normalized with 4.2 gene copies, the mean 16 S rRNA gene copy number averaged across all known bacteria^35^.

## Results

### Presumptive diagnoses

To ensure accurate labeling of our training images, we implemented a two-tiered molecular diagnostic approach combining pooled colony-level screening with individual larval microbiome analysis. For initial screening, we pooled cDNA from 10 diseased larvae and 10 healthy larvae per colony or apiary to rapidly assess pathogen presence (Table S4). We established a cycle threshold (Ct) value of 27 as our cutoff for positive infection status, with samples showing Ct values below this threshold considered infected. Healthy larval pools consistently showed Ct values above 30 for nearly all pathogens tested. While co-infections were commonly detected in field samples, we excluded co-infections of EFB with virus from our training dataset to maintain clear diagnostic categories.

**Fig. 1:**
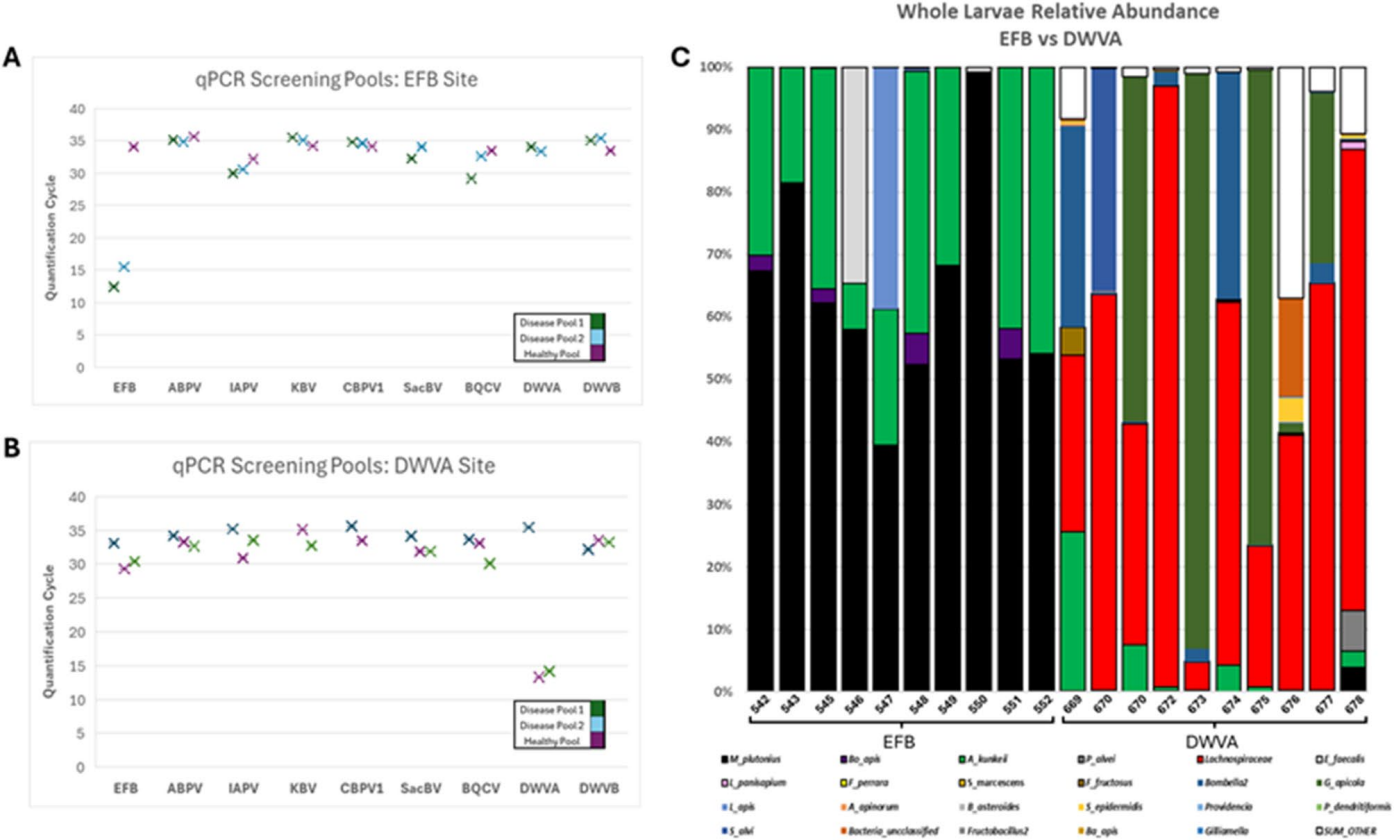
Molecular diagnostics for presumptive disease classification of honey bee larvae. (**A**) qPCR screening of pooled larvae (*n*=10 per pool) from a colony with presumptive European Foulbrood diagnosis. Three pools were tested: two of diseased larvae and one containing healthy larvae within the same apiary. Lower Ct values indicate higher pathogen loads, with Ct<27 considered positive for infection. EFB shows consistently low Ct values (~12–16) in diseased pools. (**B**) qPCR screening of pooled larvae from a colony with presumptive DWV-A diagnosis. DWV-A shows low Ct values (~13–15) in diseased pools, while healthy pools from the same colony show Ct values>30 for most pathogens. (**C**) Whole larvae microbiome composition (16 S rRNA gene relative abundance) comparing individual larvae with EFB (samples 542–552) versus DWV-A infections (samples 669–678). EFB-infected larvae show dominant *Melissococcus plutonius* (black) populations exceeding 50% relative abundance. DWV-A infected larvae display more diverse microbiota dominated by Lachnospiraceae (red) and other opportunistic bacteria, with minimal *M. plutonius* presence. This distinct microbiome signature, combined with pooled qPCR screening, enabled accurate molecular verification of disease status for training image labels.

Figure 1 illustrates our molecular diagnostic results. Figure 1A shows colonies with presumptive EFB diagnosis, while Fig. 1B displays colonies positive for DWV-A. Notably, Fig. 1C reveals the distinct microbiota signatures associated with each disease state. Larvae from samples 542–552, diagnosed with EFB, showed *Melissococcus plutonius* relative abundances exceeding 50% of the total bacterial community. In contrast, larvae infected with DWV-A exhibited microbiota dominated by opportunistic bacteria^13,14^. Individual larval microbiome analysis (Table S5) further validated our diagnostic approach. Quantitative 16 S rRNA gene analysis revealed that EFB-infected larvae carried bacterial loads approximately three orders of magnitude higher than DWV-A infected larvae, consistent with the proliferative nature of bacterial versus viral infections.

This molecular verification strategy enabled confident assignment of disease status at both colony and individual larval levels. By establishing colony-level diagnoses through pooled screening and confirming with individual larval microbiomes, we could accurately label not only individual larvae but also full frame images and multi-larvae photographs. All training images were subsequently cropped to contain 1–3 larvae and resized to either 224 × 224 or 299 × 299 pixels, depending on the model architecture requirements.

### Development of the initial disease classification model

**Fig. 2:**
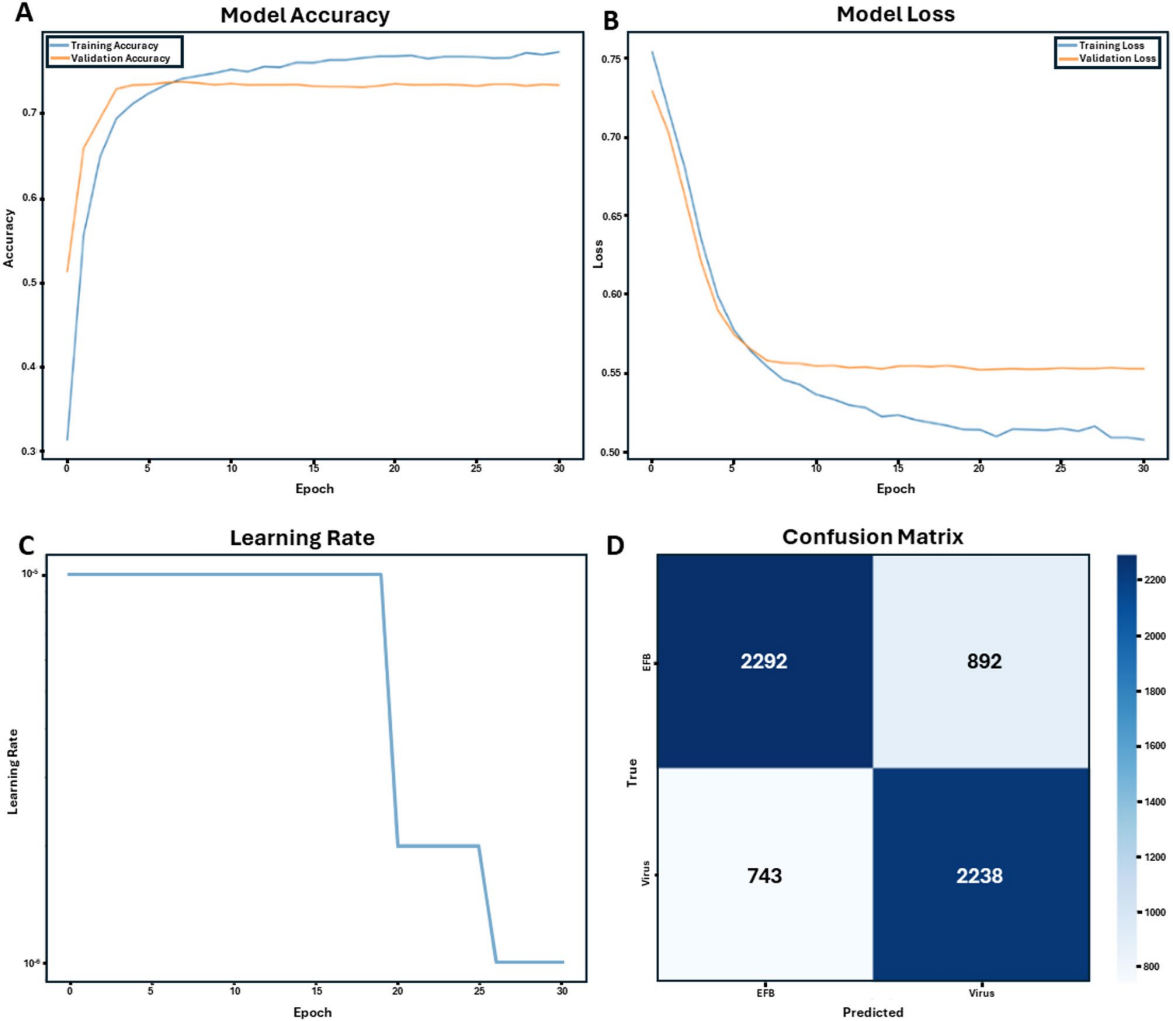
Performance metrics of the ResNet-50v2 machine learning model for honey bee brood disease classification (EFB vs. Virus). (**A**) Model accuracy curves for both training and validation datasets over 30 epochs, demonstrating the model’s learning progression. (**B**) Loss curves for training and validation sets over 30 epochs, indicating the model’s error reduction over time. (**C**) Learning rate adjustment strategy, showing step-wise decreases to fine-tune model performance. (**D**) Confusion matrix generated from testing the trained ResNet-50v2 model on an independent dataset from Illinois (2019) containing 3,184 EFB and 2,981 viral infection images. This independent evaluation demonstrates the model’s performance, correctly identifying 2,292 EFB cases and 2,238 viral infection cases, with 892 and 743 misclassifications, respectively.

Using transfer learning approaches with pre-trained deep convolutional neural networks, we developed models capable of distinguishing between European Foulbrood (EFB) and viral infections in honey bee larvae. The training dataset consisted of molecularly verified cases of EFB and viral infections, including larvae affected by Acute Bee Paralysis Virus (ABPV) and Deformed Wing Virus A and B (DWV). Training progression over 30 epochs demonstrated steady improvement in model accuracy for both training and validation datasets (Fig. 2A). The model’s learning curve showed consistent reduction in loss values throughout the training process, with both training and validation losses converging to stable values (Fig. 2B). A step-wise learning rate adjustment strategy was implemented to optimize model performance (Fig. 2C), resulting in improved convergence characteristics during later training epochs.

### Model performance and classification accuracy

Final models achieved overall accuracy rates between 73 and 88% on the training and validation datasets. When testing on a naïve dataset, analysis of the confusion matrix revealed that the model correctly identified 2,292 EFB cases and 2,238 viral infection cases, with 892 and 743 misclassifications, respectively (Fig. 2D). Performance metrics showed stronger reliability in detecting EFB (72–88% accuracy) compared to viral infections (28–68% accuracy) when tested on geographically distinct datasets not used in training.

### Visual feature analysis and model interpretation

**Fig. 3:**
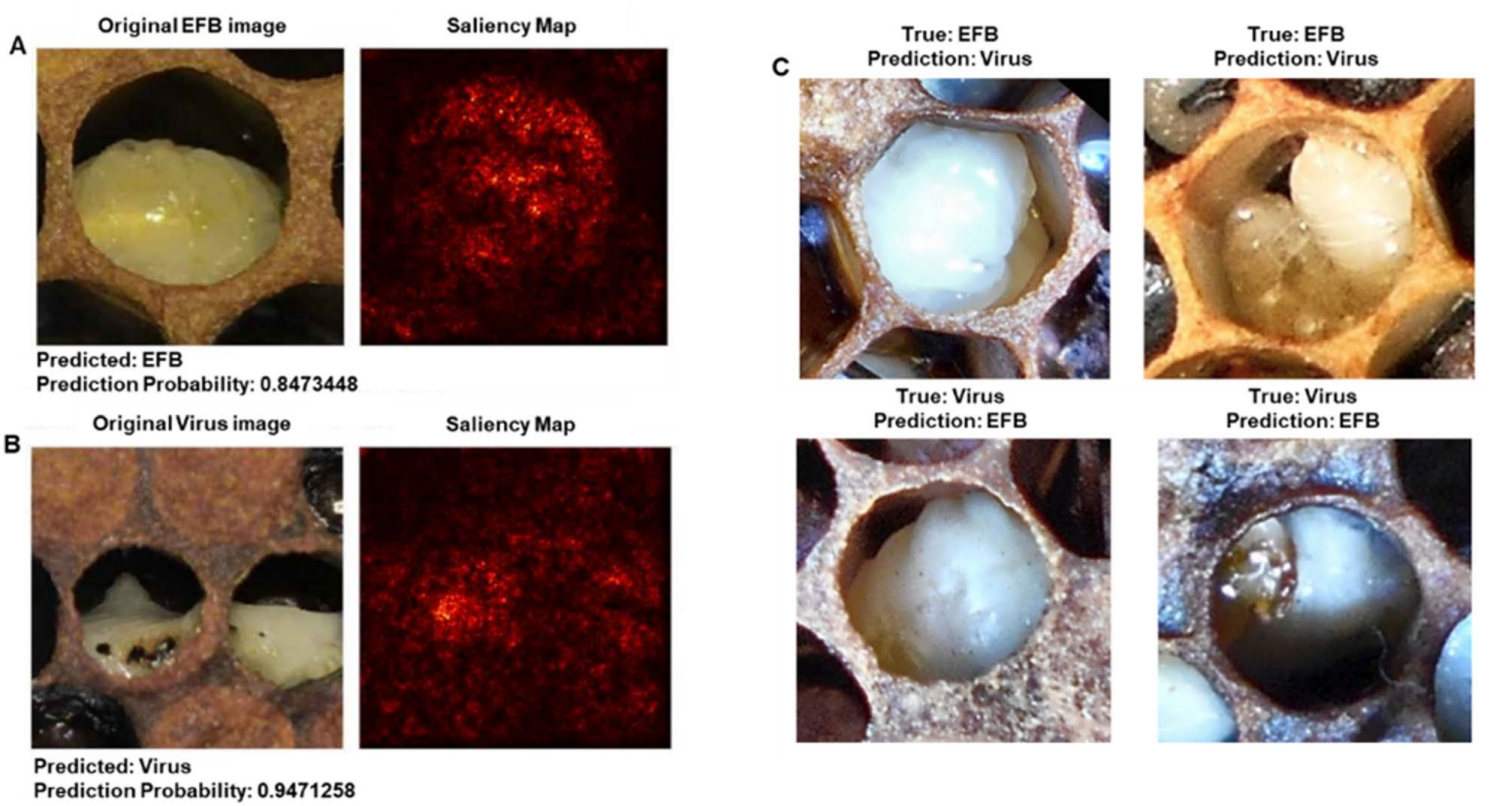
Preliminary application of explainable AI techniques to honey bee brood disease classification using ResNet-50v2. (**A**) Correctly classified European Foulbrood (EFB) image with its corresponding saliency map, generated using Grad-CAM. The saliency map confirms the model focuses on larval tissue within the cell rather than background features. (**B**) Correctly classified viral infection image with its saliency map. (**C**) Examples of misclassified images. The top row shows EFB cases misclassified as viral infections, while the bottom row shows viral infections misclassified as EFB. These preliminary visualizations demonstrate the application of interpretability tools that will be essential for future model validation. While current saliency maps confirm appropriate focus on larval tissue, more sophisticated analyses with larger, diverse datasets will be needed to identify specific diagnostic features and understand classification errors.

Saliency mapping analysis revealed the regions of importance used by the model for disease classification (Fig. 3). For correctly classified cases, the model focused on specific morphological features characteristic of each disease state. In EFB cases (Fig. 3A), the model emphasized larval tissue coloration and position within the cell, while viral infection cases (Fig. 3B) showed attention to different patterns of tissue deterioration and color changes. The saliency maps provided insight into the visual cues that differentiate these disease states, though some features like the black space in EFB cells could potentially be confounding factors in classification.

### Explainable AI: a tool for future model development

To explore the potential of explainable AI techniques in understanding model decision-making, we conducted preliminary saliency mapping using Grad-CAM^36^ on our ResNet-50v2 model (Fig. 3). While these initial visualizations confirm that the model appropriately focuses on larval tissue rather than background features, we acknowledge this represents only a basic application of interpretability methods.

## Discussion

Our study demonstrates the potential of deep learning approaches to revolutionize honey bee disease diagnostics, achieving accuracy rates of 73–88% in differentiating between bacterial and viral infections. This represents a significant advance in addressing the challenge of brood disease diagnosis, which has traditionally relied on visual inspection by experienced apiarists with variable accuracy^14^. The development of accurate, AI-driven diagnostic tools is particularly crucial given recent evidence of increasing antibiotic resistance in honey bee pathogens^10^. Our previous work has shown that inappropriate antibiotic use can disrupt the native gut microbiome, potentially increasing colony susceptibility to both bacterial and viral pathogens^37^. This is especially concerning as the honey bee microbiome plays essential roles in nutrition, immunity, and disease resistance^13,38^.

The higher accuracy in detecting European Foulbrood (EFB) compared to viral infections (72–88% vs. 28–68%) can be largely attributed to differences in training data representation. While our training dataset included viral infections from ABPV and DWV, the Illinois test dataset contained additional viral pathogens, including IAPV and BQCV, as well as DWV-A and DWV-B variants. The model’s poor performance on viral infections likely reflects this mismatch - it was asked to classify viral pathogens it had never encountered during training. This finding underscores a critical limitation of our current proof-of-concept: the viral training data did not adequately represent the diversity of viral pathogens present in field conditions. Perhaps individual larvae would be more definitive than pooled larvae, but at this early stage, the pooled approach was an attempt to distinguish EFB from virus in general. The lower accuracy in viral detection highlights the need for expanded training datasets encompassing a broader range of viral pathologies and their co-infections. Additionally, EFB may present more consistent visual symptoms across colonies compared to the variable manifestations of different viral infections. Environmental factors and strain variations that influence disease manifestation in honey bee colonies^14,17^ may further contribute to this variability.

These results highlight the urgent need for expanded and more representative training datasets. Future model development must include a comprehensive sampling of all major viral pathogens (ABPV, BQCV, CBPV, DWV variants, IAPV, KBV, LSV, SBV) as well as co-infections and novel emerging pathogens. By creating models trained on this broader spectrum of honey bee brood diseases, we can develop a diagnostic tool that truly captures the complexity of disease presentations encountered in commercial beekeeping operations.

The successful application of transfer learning in this context adds to a growing body of evidence supporting the utility of AI in apiculture. While previous studies have applied machine learning to aspects such as subspecies identification^24^ and Varroa mite detection^27^our work represents the first application specifically targeting brood disease diagnostics. The ability to accurately diagnose diseases through image analysis could significantly impact commercial beekeeping practices by enabling more targeted treatment approaches.

While our preliminary saliency mapping confirmed that the model focuses on larval tissue, we recognize that meaningful interpretability analysis requires more sophisticated approaches and larger datasets. Future applications of explainable AI techniques could reveal which specific morphological features distinguish disease states and whether AI models identify diagnostic patterns not readily apparent to human observers. Such insights will be crucial for validating model reliability and building trust with end users. The implications of this work extend beyond immediate diagnostic applications. By reducing reliance on prophylactic antibiotic treatment, this technology could help preserve the complex microbial communities essential for colony health. Furthermore, the integration of AI-driven diagnostics with molecular analyses creates new opportunities for studying the evolution of pathogen resistance and host-pathogen interactions in social insect systems.

**Fig. 4:**
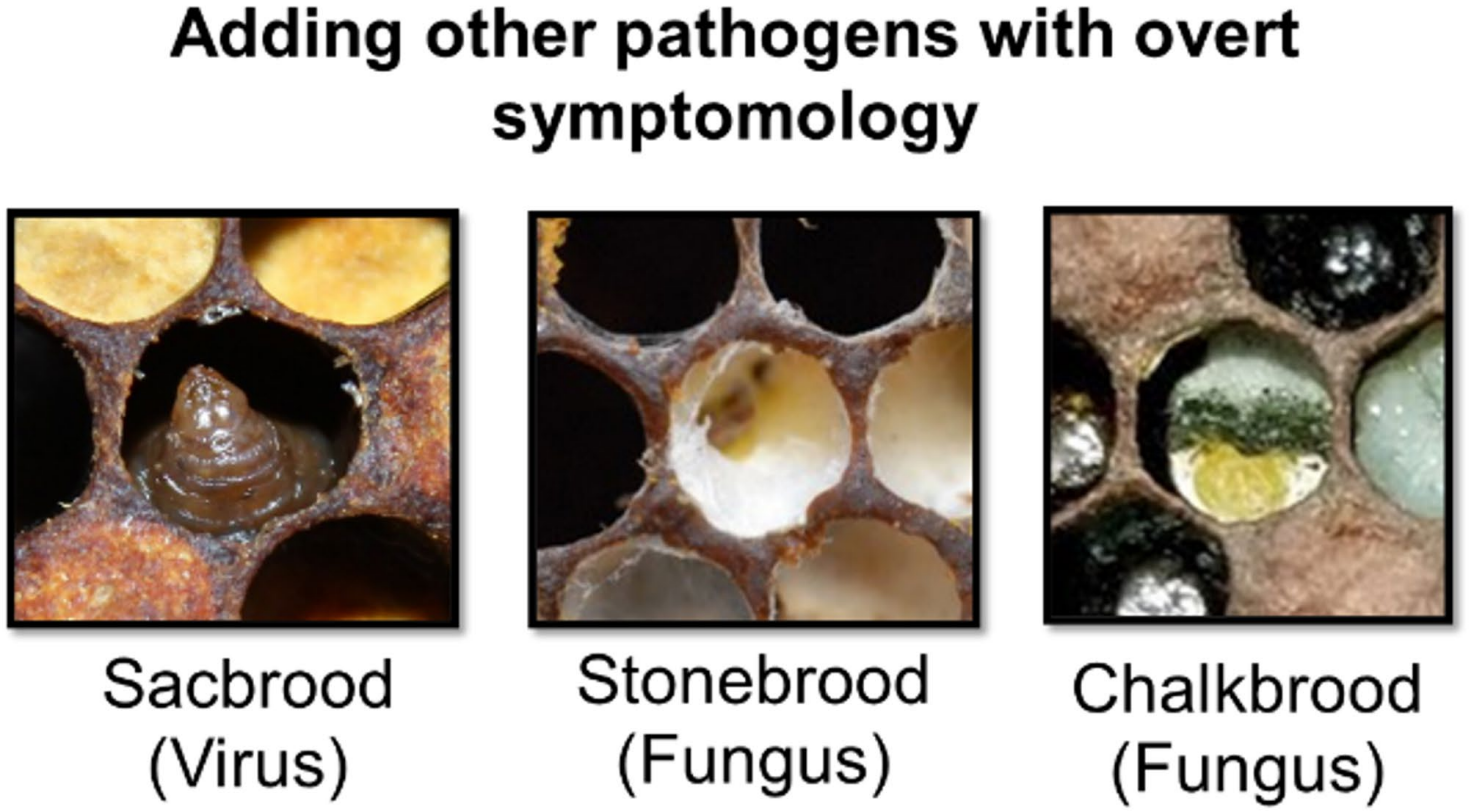
Examples of various brood disease with overt symptomology. The inclusion of these pathogens and other viruses are just one logical next step towards building a successful AI diagnosis application.

Future development should focus on expanding the training dataset to include a broader range of pathogens and their co-infections (Fig. 4), as well as integrating environmental and seasonal factors that might influence disease presentation. Our immediate next step will be to combine our Michigan training data with the Illinois test data to create a more geographically diverse training set. However, this approach will require collection of entirely new, naive datasets from additional states for unbiased model validation. This iterative process of expanding training data while maintaining independent test sets will be crucial for developing models that generalize across the diverse beekeeping regions of North America.

Our current work establishes the computational pipeline for these investigations. As we expand our dataset to include these parameters, sophisticated interpretability analyses will be essential for validating that models are learning biologically meaningful features rather than spurious correlations. This will be particularly important for building trust with beekeepers and regulatory bodies who will ultimately use these tools.

Emerging technologies such as in-hive flatbed scanners^39^ for non-destructive, long-term monitoring of honey bee brood could provide complementary data streams, enabling tracking of disease progression over time and potentially improving early-stage detection when combined with AI classification. Additionally, implementing more sophisticated interpretability methods across multiple model architectures will help identify robust visual markers of disease. The development of mobile applications could make this technology more accessible to beekeepers and apiary inspectors in the field, though successful deployment will require extensive validation across diverse geographic regions and beekeeping practices.

## Conclusion

This study demonstrates the successful development and validation of an AI-driven approach for differentiating between bacterial and viral infections in honey bee brood, achieving accuracy rates of 73–88% on training and validation data. When tested on an independent dataset, our models showed differential performance, with higher accuracy for EFB (72–88%) than viral infections (28–68%), revealing both promise and current limitations. Deep learning models can provide rapid, objective disease diagnostics, potentially reducing the inappropriate use of antibiotics in commercial beekeeping operations. The performance differences between pathogen types highlight clear directions for future improvement, particularly the need for training data that encompasses the full diversity of viral pathogens affecting honey bees.

We present this analysis not as a definitive finding, but as a demonstration of tools that will become increasingly important as we expand this technology. Future applications of explainable AI techniques could address critical questions including:

1. **Co-infection patterns**: How do models classify larvae with multiple pathogens? Which visual features dominate in mixed infections?
2. **Cross-model consistency**: Do different architectures (ResNet, Inception, Vision Transformers) identify the same diagnostic features, or do they rely on different visual cues?
3. **Temporal disease progression**: Can interpretability methods reveal how visual features change across disease stages, potentially enabling earlier detection?
4. **Novel feature discovery**: Might AI models identify subtle diagnostic features that experienced apiarists have not recognized?

Beyond the technical achievement, this work represents a significant step toward more sustainable beekeeping practices. By enabling more precise disease diagnostics, this technology could help preserve the essential microbial communities that support honey bee health while slowing the development of antibiotic resistance. The integration of artificial intelligence with traditional diagnostic approaches creates new opportunities for understanding and managing honey bee diseases across diverse agricultural landscapes. Future refinements of this technology will require iterative expansion of training datasets across geographic regions while maintaining independent validation sets. Critical next steps include incorporating more viral pathogen data, adding healthy larvae as a diagnostic class, and eventual development of mobile applications to make this tool widely accessible to beekeepers and apiary inspectors. This work establishes a foundation for implementing data science solutions in apiculture while contributing to the broader goals of agricultural sustainability and food security.

## Supplementary Information

Below is the link to the electronic supplementary material.

Supplementary Material 1

## Data Availability

The 16 S rRNA gene sequence data for larval microbiomes have been deposited in the NCBI Sequence Read Archive under BioProject accession number PRJNA1285920. Machine learning code, including training scripts and model architectures, is provided as supplementary materials. A representative sample image dataset is included in the supplementary materials to demonstrate data structure and methodology. The complete honey bee larvae image dataset and trained model weights are proprietary and currently under evaluation for patent protection by the USDA, and are therefore not publicly available. Researchers interested in collaboration or access to the full dataset should contact the corresponding authors to discuss potential data sharing agreements.
